# On the need for the development of a cancer early detection, diagnostic, prognosis, and treatment response system

**DOI:** 10.2144/fsoa-2019-0028

**Published:** 2019-11-29

**Authors:** Tobore Onojighofia Tobore

**Affiliations:** 1 Independent Researcher, San Diego, California, 92110, USA

**Keywords:** bioelectronics, bioengineering, biomarkers, biosensors, biotechnology, cancer early detection, diagnostic biomarkers, ISWEBDS, nanotechnology, personalized/precision medicine, predictive biomarkers, prognostic biomarkers, targeted therapy, wearables

## Abstract

Cancer is the second leading cause of noncommunicable disease deaths in the world. In 2018, there were over 18 million new cancer cases and approximately 10 million people died from the disease globally. In 2019, almost two million new cases of cancer will be diagnosed in USA and over 600,000 people are expected to die from the disease. The incidence of cancer is expected to rise because of lifestyle changes and a rapidly aging population. Evidence suggests that early detection is critical to reducing cancer morbidity and mortality. In this paper, the development of an integrated smart wearable and biomarker detection system is proposed to help reduce cancer morbidity and mortality. The potential benefits and limitations of the system are discussed.

## Lay abstract

Cancer is one of the leading causes of death in the world. Evidence suggests that its incidence will continue to rise in the future because of lifestyle changes and a rapidly aging population. There is currently no cure for the disease and the best way to reduce its incidence and morbidity is to detect it early. In this paper, an integrated smart wearable and biomarker detection system to help in the early detection, prognosis, diagnosis and treatment of cancer is proposed.

In 2015, cancer was the second leading cause of noncommunicable disease deaths in the world [1]. In USA, almost two million new cases of cancer will be diagnosed and approximately 610,000 people are expected to die from the disease in 2019 [2]. Globally, over 18 million new cancer cases were identified and approximately 10 million people died from the disease in 2018 [3]. By 2020, the annual US cancer cases are predicted to increase among men by 24% (>1 million cases) and by about 21% among women (>900,000 cases) [4]. Factors such as tobacco smoking, urbanization, pollution, diet, better medical services and a rapidly aging population have been theorized to be responsible for this explosive cancer incidence [4,5].

Although there has been little progress in reducing new cancer cases, significant progress has been made in prevention and treatment measures, resulting in reduced cancer mortality [6]. From 2006 to 2015, the cancer death rate declined by approximately 1.5% annually in both men and women and from 1991 to 2015 the combined cancer death rate dropped steadily by a total of almost 30%, translating to approximately 2.5 million fewer cancer deaths [6]. However, this progress comes at a huge financial cost. Estimated national expenditure for cancer care in USA in 2010 was approximately US$130 billion and in 2020 it is projected to be almost US$160 billion [7,8]. Across the world, the cost of cancer is equally high. In 2009, cancer costs the European Union (EU) €126 billion (US$146 billion) [9]. In China, the total payments on cancer treatments were estimated to be 221.4 billion RMB (US$31 billion) in 2015 [10]. In future years, costs are likely to increase as the population ages. The financial burden at the individual level is equally high. Indeed, the exorbitant cost of cancer drugs and treatment, whose value is uncertain, is a huge problem that puts cancer sufferers and their families in significant financial distress [11–13].

Therefore, the need for a better approach to reduce the incidence and morbidity from cancer cannot be overemphasized.

## Early detection is critical

Cancers are often found by symptomatic presentation, which may manifest as a breast lump, rectal bleeding, persistent cough, lymphadenopathy and weight loss [14]. Indeed, in many cases, the patient remains asymptomatic and thus no medical treatment is sought. Many patients are unaware of or simply ignore the symptoms of cancer because of poor health literacy, the financial cost of hospital visits (particularly in the USA), cultural attitudes toward seeking medical care, fear of a cancer diagnosis and challenges with navigating the healthcare system [15]. By the time symptoms become apparent and the patient seeks medical help, it may be out of reach of available clinical treatment [16]. Late detection of cancer makes treatment difficult because of progressive advancement in disease stage and metastasis.

Physicians contribute to the problems of late detection by failing to recognize important signs and taking further investigational steps to ensure that cancer is eliminated as a potential cause of the patient’s symptoms [15]. Although early detection programs have increased, particularly in the developed world, such programs typically rely on symptomatic presentation. However, early cancer symptoms can be nonspecific and can be easily confused for other conditions [15], contributing to the delay in diagnosis and the progression of the disease.

## The way forward: integration of biosensors, biomarkers & wearables

Biological markers (biomarkers) can be defined as cellular, biochemical or biological substances that can be measured and evaluated objectively as indicators of pathogenic processes, normal biological processes or pharmacological responses to a treatment regime [17]. Biomarkers have also been described to include tools and technologies that can aid in the prediction, cause, diagnosis and pharmacological responses or outcome to a therapeutic intervention (progression or regression of disease following treatment) [18]. Biomarkers have been shown to be useful and used for decades in the prediction, diagnosis and management of different diseases including immunological, metabolic and genetic disorders, neurological and cardiovascular diseases, infections and cancer [19–23]. Advances in molecular biology and laboratory technology have expanded the use and feasibility of applying biomarkers, particularly in clinical trials, analytic epidemiology and the management of different diseases [18].

Biosensors typically consist of a biological sensing element (enzymes, antibodies, DNA/RNA, tissues or other biomolecules) and an electrochemical transducer [24], are analytical tools which have applications in detecting biomarkers of different diseases including neurological, cancer, cardiovascular and immune disorders [25–30]. Electrochemical biosensors are widely developed and have broad applications including in environmental, agricultural, biological, biomedical, biotechnological, clinical and medical diagnostics and health monitoring [31,32].

Wearable technology usage is increasing in USA and around the world. Indeed, one in six consumers in USA currently uses wearable technology (smartwatches or fitness bands) [33]. Research indicates that approximately 19 million fitness devices were sold in 2016, and a whopping 110 million were forecasted to be sold in 2018 [34]. The rise of wearable technology provides an opportunity to radically transform the field of healthcare. Wearables could give patients direct access to personal analytics, which can contribute to their wellbeing, promote preventive care and assist in the treatment of disease [33]. Also, it provides a new opportunity to apply machine learning to patient health information and immediately inform patients and clinicians when a health problem arises [35].

An integrated smart wearable and biomarker detection system (ISWEBDS), consisting of electrochemical biosensors to detect clinically relevant biomarkers and transmit the data to a smart wearable, will provide a novel and powerful tool that can revolutionize healthcare. It could help the treatment of chronic diseases, like cancer, by enhancing early detection, diagnosis, prognosis and the understanding of pharmacological responses or outcomes to therapeutic intervention. These electrochemical biosensors will be implanted in parts of the body most prone to carcinogenesis (colon, breast, esophagus, lung, stomach, cervix and prostate or anywhere in the body considered clinically useful) and be used to monitor the microenvironment for targeted cancer biomarkers in real time.

**Figure 1.**
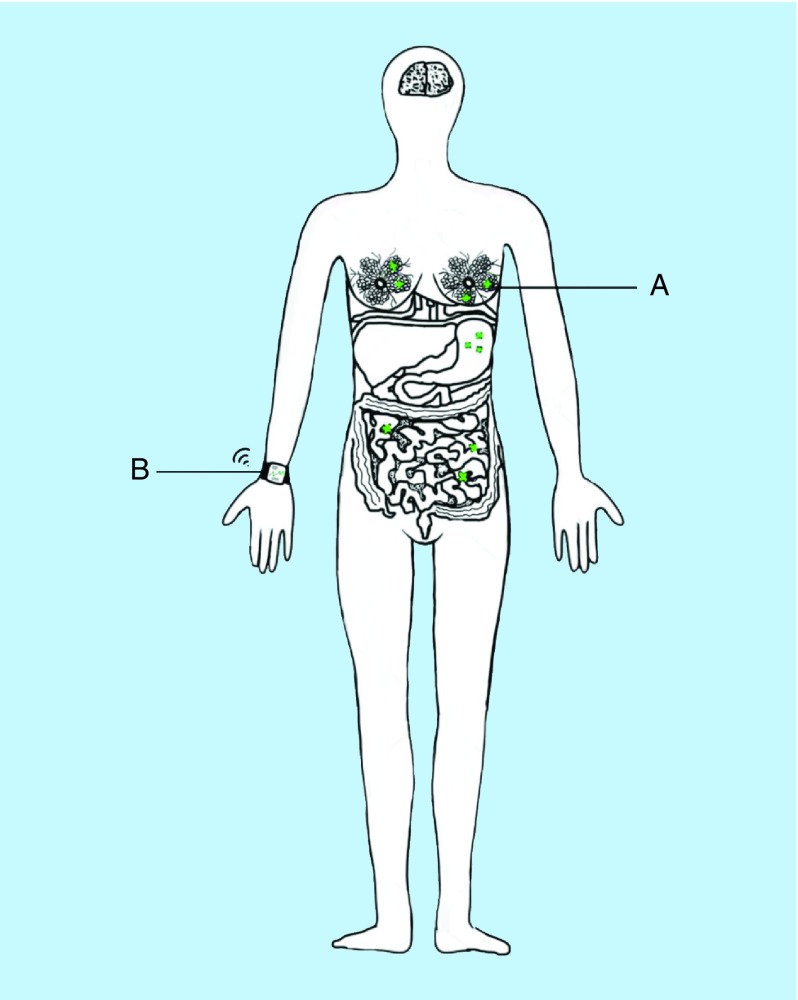
Integrated smart wearable and biomarker detection system. **(A)** Biosensors implanted in the body. **(B)** Smart wearable receiving signals from biosensors.

Figure 1 below gives an overview of the system. The biosensors will sense, collect, interpret and send signals to patients via the wearable when defined levels of a target cancer biomarker are detected. This information can be accessed through a smartphone, computer, tablet or the cloud. A dedicated application connected to the wearable can be accessed by both the patient and a clinician who can interpret and translate the data to clinically valuable or actionable information.

Multiples lines of evidence indicate that oxidative stress (OS) biomarkers can be used to predict different types of cancers [36–40]. 8-oxo-7,8-dihydro-2’-deoxyguanosine (8-oxodG) [35] is an example of an OS biomarker used in predicting the risk of lung and breast cancers. Cytogenetic biomarkers, which are used to assess polymorphisms that alter the frequency of chromosomal aberrations, sister chromatid exchanges and micronuclei in peripheral lymphocytes [41,42], play a critical role in predicting the risk of different cancers. Circulating biomarkers, which include circulating DNA and micro-RNA (miR; e.g., miR-375, miR-141, miR-378* and miR-409-3p) are effective for the prediction, early detection, prognosis and treatments of different types of cancers [43–47]. Several protein biomarkers including CA-125α, fetoprotein, human chorionic gonadotropin, LDH and carbohydrate antigen 19-9 (CA19-9) have also been shown to be effective for the prediction, early detection, prognosis and treatments of different types of cancers [48–51]. Table 1 below shows different cancer biomarkers and their uses.

**Table 1.** Different types of cancer biomarkers and their uses.

Types of cancer biomarkers	Uses	Types of cancer
Cytogenic biomarkers	Used to evaluate genetic exposure to toxic carcinogenic or mutagenic agents [42] An example includes PI3K pathway mutation assessment for head and neck squamous cell carcinoma and γ-H2AX formation for prediction, prognosis, diagnosis and treatment response in different human cancer cell lines, premalignant lesions and solid tumors [52–54]	– Lung cancers – Brain cancers – Renal cancers – Pancreatic cancers
Circulating biomarkers	Circulating tumor DNA, circulating tumor cells or circulating microRNA in blood and other body fluids [43,55]	– Colorectal cancer – Pancreatic cancer – Breast cancer – Head and neck cancer – Small intestine cancer – Endocrine tumors – Prostate cancer – Osteosarcoma – Brain tumors – Glioblastoma
Protein biomarkers	Assess for specific breast cancer protein biomolecules in nipple aspirate fluid secretome, urine samples or in other body fluids [56] **Diagnostic**: KIT protein for gastrointestinal stromal tumors [54] **Predictive/prognostic:** 1. CA-125 for ovarian cancer 2. α-fetoprotein, human chorionic gonadotropin 3. LDH in testicular cancer 4. Prostate-specific antigen for prostate cancer 5. Estrogen receptor tissue marker in breast cancer 6. Carbohydrate antigen 19-9 (CA19-9) pancreatic and colorectal cancer **Treatment/pharmacological response**: 1. Prostate-specific antigen for prostate cancer treatment 2. Carbohydrate antigen 19-9 (CA19-9) for pancreatic cancer 3. In gastric or gastro‐oesophageal junction cancers, HER2 levels can be used in selecting patients for trastuzumab treatment [54] 4. Carcinoembryonic antigen levels in postoperative monitoring of stage II and III gastrointestinal cancer patients for surgical resection or systemic treatment [54]	– Breast cancer – Urothelial cancer – Lung cancer – Colon cancer
Oxidative stress biomarkers	Biomarkers indicating alterations in redox homeostasis toward oxidizing conditions	– Breast cancer – Lung cancers – Pancreatic cancer

## Discussion

More than any other factor, early detection (presymptomatic stage) is critical to arresting cancer due to progressive advancement in disease stage and metastasis. ISWEBDS could play a crucial role in early detection during the presymptomatic stage, reducing the morbidity, mortality and financial burden of the disease. It could play a key role in cancer prevention by detecting precursors of cancers or precancerous lesions, reducing the incidence of the disease. It could also be extremely useful in successful cancer treatment by providing insight into pharmacological responses or outcomes to therapeutic interventions. Indeed, changes in circulating DNA mutation patterns during cancer treatment typically indicate the appearance of resistant clones and it can be used to alter treatment strategy [55,57].

ISWEBDS can overcome the clinician’s dependence on patient self-reporting for clinical decisions and it can cut out the need for regular onsite screening, which is necessary for detecting clinically relevant biomarkers. Thus, it will help in reducing cancer treatment costs by cutting expenditure on the regular conduct of laboratory diagnostic tests. ISWEBDS should make it easy to collect more reliable and responsive ratio-scaled outcome measures from patients remotely and in real time, reducing onsite follow-up and potentially increasing clinical trial recruitment and retention due to reduced cost and burden of travel.

Also, as ISWEBDS sends information to patients about their health status, it is likely to make them more informed about their activities and how it affects their health, causing them to make healthier choices. Indeed, research indicates that a well-informed patient is motivated to engage in healthy behavioral changes [58].

There are, however, some problems that must be overcome to make ISWEBDS a reality. In the last two decades, there have been rapid developments in the field of wireless body area networks [59], wearables, biosensors and bioelectronics [60–64]. In addition, although recent developments in biosensor technology have significantly improved the sensitivity of biomarkers in the early stages of cancer [65], challenges remain in biosensor technology, particularly in integration [66,67]. The invasive nature of the system is another limitation and biodegradable biosensors that are durable and programmable remotely will be the most effective way to make them work. Inflammation and subsequent OS from biosensor placement around tissues could lead to carcinogenesis. Biomarker sensitivity and specificity [68] is another problem that must be addressed to make the system a reality. Integrating the data from the biosensors to the wearable and knowing the precise level of a biomarker that should trigger an alert are challenges that must be overcome through research. The system might be costly, at least initially, making it out of reach for the poor, who might benefit the most from it. Also, placing biosensors in certain parts of the body, particularly in the lungs and the brain, is perhaps impractical, indicating that the system might be limited to certain areas of the body, potentially reducing its ability to detect cancers early in some parts of the body. Overdiagnosis, a key feature of both early detection and preventive screening [69], could also be a problem with the system. Table 2 below summarises the advantages and challenges of developing ISWEBDS.

**Table 2.** Advantages and challenges of integrated smart wearable and biomarker detection system.

**Advantages**
1. Early detection of cancer allowing for proper intervention and care and, reducing the mortality and morbidity of the disease 2. Aid in the prevention of cancers by detecting precancerous lesions 3. Used to predict treatment response and to alter treatment course, improving outcomes and saving lives 4. Overcomes clinician’s dependence on patient self-reporting for clinical decisions and cuts out the need for regular onsite screening necessary for detecting biomarkers 5. Since it sends information to patients about their health status, it is likely to make them more informed about their activities and how it affects their health, causing them to make healthier choices
**Challenges**
1. Specificity and sensitivity of biosensors to capture the targeted biomarkers 2. Individual variability of biomarker levels in the presence of cancer or in response to cancer treatment 3. Confounding results from biomarkers due to failure to identify factors that may alter the measurement of the biomarker including weight, age, gender, diet, other metabolic factors, and laboratory kits used 4. Engineering difficulty in transferring the biosensor capture information to the smart wearable 5. The cost of developing new biomarkers is high and this might be a problem, making the system out of reach for the most vulnerable people 6. Exact location to place the biosensor and problems of inflammation, pain and oxidative stress from tissue damage 7. The invasive nature of the system might limit its adoption
**Strategies to improve functionality**
1. Investment in research to develop better biosensors that are highly sensitive and specific to certain biomarkers 2. Better collaboration between clinicians, funding agencies and the biotech industry 3. Subsidies and incentives from the government to spur research and development in biosensors, biomarkers and their integration 4. Development of biodegradable biosensors that will not induce inflammation and related problems 5. Development of less invasive methods of installing, replacing or removing biosensors

## Conclusion

The future of effective cancer treatment and management profoundly hinges upon the use of innovative methods, which will assist clinicians in disease management. An integrated system such as ISWEBDS will maximize the utility of biomarkers and the addition of biosensors and wearables will help in capturing labile biomarkers (biomarkers that are are liable to change or alteration and thus difficult to capture) in the cancer microenvironment. In addition, ISWEBDS could quickly determine the best treatment options, which is most likely to be successful reducing the problem of drug resistance. ISWEBDS can transform the problem of cancer by not only aiding in early detection, prevention, prognosis, recurrence and the prediction of treatment efficacy but also doing so at a fraction of the overall current cost and with great convenience for both patients and clinicians. It has the potential to revolutionize healthcare in this century by expanding the capabilities of the healthcare system, improving diagnostics and monitoring and the participation of patients in their wellbeing. However, significant collaboration would be necessary between clinicians, engineers and the biotechnology industry to make it a reality.

Finally, early cancer detection does not necessarily translate to clinical benefits for patients and emphasis must equally be placed on early effective treatment following diagnosis to reduce the morbidity and mortality from the disease. In addition, although the article focuses on the use of ISWEBDS on cancers, it can be applied for the diagnosis, prediction, prognosis and treatment of any chronic disease.

## Future perspective

The treatment of cancer currently involves different approaches including chemotherapy, radiotherapy, surgery and pharmacogenomics. Much effort has been expended on finding an outright cure to no avail and evidence suggests such a cure may never materialize in the near future. Consequently, the best way forward lies in early detection and ISWEBDS, if successfully developed, can play an important role in reducing the incidence and burden of cancers.

**Executive summary**

- Cancer is one of the leading causes of noncommunicable disease deaths in the world.
- Multiple lines of evidence indicate that early detection is critical to reducing the incidence and burden of the disease.
- In this paper, an integrated smart wearable and biomarker detection system (ISWEBDS) to help in the early detection, prognosis, diagnosis and treatment of cancer is proposed.
- ISWEBDS has the potential to revolutionize healthcare in this century by expanding the capabilities of the healthcare system, improving diagnostics and monitoring and the participation of patients in their wellbeing.
